# A randomised controlled trial of acceptance and commitment therapy (ACT) for psychosis: study protocol

**DOI:** 10.1186/1471-244X-14-198

**Published:** 2014-07-11

**Authors:** Neil Thomas, Frances Shawyer, David J Castle, David Copolov, Steven C Hayes, John Farhall

**Affiliations:** 1 School of Health Sciences, Swinburne University, Hawthorn, Victoria 3122, Australia; 2 Monash Alfred Psychiatry Research Centre, Melbourne, Victoria 3004, Australia; 3 Department of Psychiatry, Monash University, Clayton, Victoria 3800, Australia; 4 School of Psychological Science, La Trobe University, Melbourne, Victoria 3086, Australia; 5 Department of Psychiatry, University of Melbourne, Parkville, Victoria 3052, Australia; 6 St Vincent’s Hospital Mental Health, Fitzroy, Victoria 3065, Australia; 7 Office of the Vice-Chancellor and Discipline of Psychiatry, Monash University, Clayton, Victoria 3800, Australia; 8 Department of Psychiatry, University of Melbourne, Parkville, Victoria 3010, Australia; 9 Florey Institute of Neuroscience and Mental Health, Parkville, Victoria 3052, Australia; 10 Department of Psychology, University of Nevada, Reno, Nevada 89557, USA; 11 NorthWestern Mental Health, Royal Melbourne Hospital, Melbourne, Victoria 3050, Australia

**Keywords:** Randomised controlled trial (RCT), Psychosis, Schizophrenia, Auditory hallucinations, Delusions, Positive symptoms, Negative symptoms, Acceptance and commitment therapy (ACT), Psychological therapy, Befriending

## Abstract

**Background:**

Cognitive behavior therapy for psychosis has been a prominent intervention in the psychological treatment of psychosis. It is, however, a challenging therapy to deliver and, in the context of increasingly rigorous trials, recent reviews have tempered initial enthusiasm about its effectiveness in improving clinical outcomes. Acceptance and commitment therapy shows promise as a briefer, more easily implemented therapy but has not yet been rigorously evaluated in the context of psychosis. The purpose of this trial is to evaluate whether Acceptance and Commitment Therapy could reduce the distress and disability associated with psychotic symptoms in a sample of community-residing patients with chronic medication-resistant symptoms.

**Methods/Design:**

This is a single (rater)-blind multi-centre randomised controlled trial comparing Acceptance and Commitment Therapy with an active comparison condition, Befriending. Eligible participants have current residual hallucinations or delusions with associated distress or disability which have been present continuously over the past six months despite therapeutic doses of antipsychotic medication. Following baseline assessment, participants are randomly allocated to treatment condition with blinded, post-treatment assessments conducted at the end of treatment and at 6 months follow-up. The primary outcome is overall mental state as measured using the Positive and Negative Syndrome Scale. Secondary outcomes include preoccupation, conviction, distress and disruption to life associated with symptoms as measured by the Psychotic Symptom Rating Scales, as well as social functioning and service utilisation. The main analyses will be by intention-to-treat using mixed-model repeated measures with non-parametric methods employed if required. The model of change underpinning ACT will be tested using mediation analyses.

**Discussion:**

This protocol describes the first randomised controlled trial of Acceptance and commitment therapy in chronic medication-resistant psychosis with an active comparison condition. The rigor of the design will provide an important test of its action and efficacy in this population.

**Trial registration:**

Australian New Zealand Clinical Trials Registry: ACTRN12608000210370. Date registered: 18 April 2008

## Background

### Cognitive behavior therapy (CBT) for psychosis

The continued prevalence of medication-resistant psychotic symptoms has prompted an interest in developing psychological treatments as an adjunct to medication to improve symptoms. Cognitive behavior therapy (CBT) has been the dominant therapy approach applied to psychosis, aiming to reduce the distress and disability associated with psychotic symptoms such as hallucinations and delusions. Whilst CBT for Psychosis (CBTp) includes a number of facets [1,2], the core intervention characterising this approach is belief modification (cognitive restructuring) – the ‘C’ in CBT [3]. This involves modifying the content of anomalous beliefs identified as being associated with distress and dysfunction such as delusions and other symptom-related cognitions that may lead to distress, e.g. the belief that hallucinated voices have the power to harm the patient. The major techniques for weakening such beliefs include challenging the evidence for them, generating alternative explanations, identifying irrational or inconsistent elements and constructing reality testing experiments [4,5].

The efficacy of CBTp for symptom improvement in schizophrenia initially showed considerable promise. Several early meta-analyses reported large and important clinical effects [6-8] providing key evidence that CBTp was an effective treatment for positive psychotic symptoms. This led to CBTp becoming an established evidence-based treatment for residual psychotic symptoms [1,9] and it has been recommended for routine provision in clinical practice guidelines now for many years [10-12]. With the advent of more rigorous trials and further meta-analyses however, the initial optimism about the impact of CBT has become increasingly cautious [3,13]. Recent reviews have concluded that CBT has only a small effect on symptoms [14,15] and questioned its advantages over other less complex therapies [16,17], although this has also been vigorously debated (e.g., [18,19]).

Even so, CBTp is limited by being a challenging therapy to deliver. Around half the patients who receive CBTp in clinical trials fail to attain clinically significant benefits in symptoms [5], suggesting this approach is only suitable for some patients. Failure to respond has been associated with pre-therapy measures of resistance to considering alternatives to delusions [20], denying any possibility of being mistaken [21], and the patient failing to engage with the therapist’s model of reality during the therapy process [22]. These observations suggest that the partial effectiveness of existing psychological treatment arose from some patients not being amenable to the process of belief modification which is so central to CBT. Even for those patients who are amenable to cognitive restructuring, modifying psychotic beliefs is usually a slow and difficult endeavour [23]. In order to avoid the risk of psychological reactance – a defensive restitution or even strengthening of the belief [24,25] – cognitive restructuring must proceed slowly and gently, and only once several sessions have been spent building up a strong therapeutic relationship [4,5,23]. A minimum duration of therapy of six months is recommended [10], with briefer interventions producing changes in mood symptoms but failing to impact upon psychotic symptomatology [10,26]. In addition, because this process is highly complex, it requires a high level of skill in CBT. Accordingly, some trials have observed poorer outcomes with less experienced therapists [27,28].

Thus, in spite of the initial apparent success of CBTp, there remains much room to improve the psychological management of psychotic symptoms. In particular, approaches that are less reliant upon belief modification to bring about therapeutic change may prove to be briefer, more effective, cost-effective, and more readily disseminated into routine practice.

### Acceptance and commitment therapy (ACT) for psychosis

Acceptance and commitment therapy (ACT) is a manualised psychological treatment [29,30] with a clear theoretical basis [31] that shows promise as an alternative approach to psychological intervention in psychosis. According to the ACT model, psychological problems develop through the inappropriate or unhelpful regulation of behavior through language processes leading to psychological inflexibility in relation to environmental contingencies [32]. Rather than effecting change through modification of belief content, ACT aims to reduce the extent to which beliefs and other symptoms dominate conscious experience and behavior. More specifically, treatment of symptoms is not focused on their removal, but on taking them less literally and disrupting their link with behavior. ACT uses experiential exercises, illustrative metaphors and behavioral tasks in order to effect change, with logical analysis having a relatively minor role [33]. Although ACT is often described as a variant of CBT, the overlap in therapeutic elements is small [1].

As the name suggests, ACT has two broad components. In the Acceptance component, methods cultivating cognitive defusion, contact with the present moment and self as the observer of experience help the individual recognise and dispassionately observe symptoms and associated reactions, rather than believing and acting on them. The Commitment component emphasises the articulation of personal values and goals, and seeks to minimise the effects of symptoms on achieving those goals in behavioral terms. At its core, ACT is a behavioral treatment [29,30], grounded in producing functional change.

Two limited RCTs have tested the application of ACT to acute psychosis with surprisingly powerful outcomes. Bach and Hayes [34] assessed the impact of a brief version of ACT on symptoms in a population of 80 inpatients with positive psychotic symptoms. Rate of rehospitalisation was used as a concrete and objective measure of the negative functional impact of symptoms. In contrast to CBTp, which involves an average of 20 sessions [35], treatment involved just four 45-minute sessions. The authors found that, compared with a treatment-as-usual control group, participants in the ACT group had half the rate of rehospitalisation (20% vs 40%) over a follow-up period of four months.

Gaudiano and Herbert [36] conducted a second trial of ACT for psychosis, providing approximately three hours of ACT to inpatients with schizophrenia and implementing an enhanced treatment-as-usual comparison condition. This trial additionally examined outcomes using validated symptom measures including the Brief Psychiatric Rating Scales (BPRS). Although there were no overall group differences on the BPRS from pre-test to follow-up, half of the participants randomised to receive ACT improved two standard deviations or more on the BPRS, compared with only about 10% of participants receiving usual ward-based therapy. In terms of functional change, participants in the ACT group reported significantly less social disruption from their symptoms compared to controls. Rehospitalisation rates showed a similar trend to the Bach and Hayes trial (28% *vs* 45%), although this difference failed to reach significance in this smaller sample (*n* = 40). However, a recent analysis of pooled data from both trials supported the original reduced rate of rehospitalisation, which was mediated by reduced symptom believability [37].

Our group earlier conducted an RCT (*n* = 43) of a 15-session psychological intervention for medication-resistant command hallucinations called “Treatment of Resistant Command Hallucinations (TORCH)”, which incorporates elements of ACT alongside CBTp [38]. While the results showed no strong evidence that the combined ACT-CBT treatment targeting command hallucinations was superior to the Befriending control condition, the *n* of 43 in this trial was considerably below what would now be considered advisable based on effect sizes published after the trial’s completion [39]. When we examined the pattern of within group findings and conducted comparisons with a waitlist control, results suggested that both interventions had benefit with a different pattern of outcomes observed across the two conditions. While Befriending was primarily effective in the short-term for distress [38], TORCH showed both short and longer-term benefit for a broader range of outcomes including illness severity.

The TORCH study was rigorously conducted including blinding and an active control. Notwithstanding its low power, outcomes were in line with the more recent evaluations of CBTp indicating no substantial benefit for symptoms compared to other psychological treatments including those which may be less sophisticated such as Befriending. However, given this trial involved a combined CBT-ACT treatment targeted to command hallucinations it is not possible to draw conclusions about the efficacy of ACT alone and also its efficacy in relation to psychotic symptoms in general. In contrast, while the initial trials of pure ACT for psychosis were positive they were not blinded and they did not have an active comparison condition. Thus, there is a need to conduct a rigorous trial of pure ACT for psychosis to test whether the promise shown in initial trials holds under more stringent conditions.

### Aim, hypothesised outcomes and mechanisms of change

The aim of this study was to test the efficacy of ACT for psychosis in a sample of community-residing patients with chronic medication-resistant psychotic symptoms by comparison with an active comparison condition. The study of ACT in this population is important because they represent the majority of service users living with psychosis. In doing so, we hypothesised a number of effects of ACT on specific symptom-related outcomes and processes that would be consistent with the ACT model.

As with CBT, ACT does not aim to eliminate psychotic symptoms, but to reduce resultant distress and disability to enable better quality of life. Both hallucinations and delusions appear to be multidimensional phenomena, in which different dimensions may show significant variation between individuals and over time, e.g., frequency, degree of preoccupation and degree of conviction in content, as well as resultant distress and impact upon behavior [40,41]. The dimensions of preoccupation and conviction appear particularly important in determining levels of disability and subjective distress. Indeed, in the general population, beliefs with the same themes as delusions may be held in attenuated form without interfering with social functioning or being found significantly distressing [42,43]. The dimensions of preoccupation and conviction appear more important than content itself in distinguishing patients with schizophrenia from non-patients who hold unconventional beliefs [42,43]. The cognitive defusion techniques used in the Acceptance component of ACT provide an avenue for reducing the level of preoccupation and conviction with psychotic symptoms, but, in contrast to CBT, without requiring modification of the content of beliefs themselves. The ACT model proposes that preoccupation with distressing internal experiences such as psychotic symptoms is maintained by the paradoxical effect of repeatedly engaging in unsuccessful attempts to avoid and suppress them [29,30]. The Acceptance component of ACT involves exercises to help the person recognise the futility of trying to directly suppress and control such experiences, combined with teaching skills in mindful observation of experiences in a detached manner without attempting to change or judge them. The use of defusion techniques is associated with increased tolerance of experimentally induced pain, as opposed to distraction or no instruction [32], suggesting that this is applicable to aversive internal experiences in general. In addition, there is evidence that defusion techniques are particularly effective in reducing repetitive cognitive processes such as depressive rumination, anxious worry and persistence of obsessive thoughts (e.g., [32,44]). These findings suggest that such techniques may also be helpful for reducing preoccupation with distressing delusions [45]. Further, there is a strong argument that distancing and acceptance techniques may also be specifically applicable to preoccupation with auditory hallucinations given their ‘verbal’ form, their compelling salience, and the propensity of voice hearers to be drawn into reacting to the literal content [46,47]. In support of this contention, several strands of research have found acceptance strategies to be helpful in managing voices (see [39] for review).

Defusion techniques are also hypothesised to have an impact on conviction related to both delusional beliefs and hallucinatory content. Bach and Hayes [34] found reduced ratings of the ‘believability’ of delusions and voice content post-treatment relative to controls, and Gaudiano and Herbert [48] found that changes in rated believability of voice content predicted reduction in distress, independent of changes in frequency of hallucination.

Although ACT may lead to reductions in preoccupation and conviction with psychotic symptoms, thereby impacting on resultant distress and disruption to life, it is unlikely to have an effect on how often symptoms are experienced. In the Bach and Hayes [34] and Gaudiano and Herbert [36] trials, no differences were observed in reported frequency of symptoms post-treatment between participants receiving ACT and treatment-as-usual. However, a noteworthy paradoxical effect was observed in the Bach and Hayes trial: relative to controls, participants in the ACT group were *less* likely to report that symptoms had completely remitted. Furthermore, those ACT participants who reported still experiencing symptoms had better outcomes in that they were significantly less likely to be readmitted. This paradoxical association of fewer reports of symptom remission with otherwise positive outcomes compared with controls may be explained by control participants being more likely to deny symptoms with the acceptance component of ACT being protective against this [34]. Denial of the psychotic experience is associated with a “sealing over” recovery style [49]. Those who seal over tend to isolate the psychotic experience and are disinterested in any exploration of symptoms [49]. In contrast, those with an “integrative” recovery style are curious to understand and explore their illness. This study will therefore examine whether ACT results in reduced sealing over of psychotic experiences and determine whether this accounts for any paradoxical increase in symptom reporting. It is hypothesised that if any paradoxical increase in the number of symptoms reported occurs, this will be mediated by recovery style.

**Figure 1:**
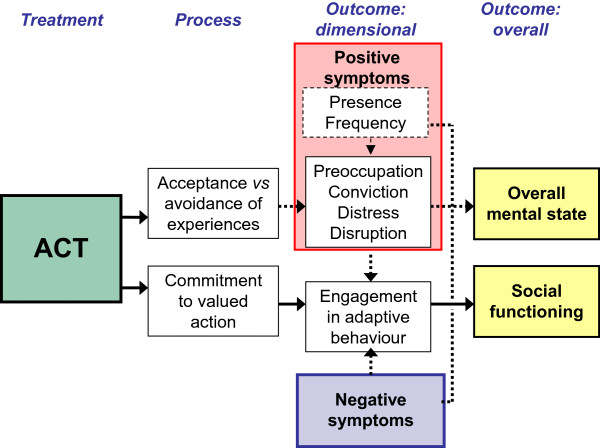
Hypothesised mechanisms of action for ACT.

The Commitment component of ACT involves exercises to help the patient clarify their personal values and encouraging commitment to take action in line with these values. It is expected that this will lead to adaptive behavioral change, reducing the impact of negative symptoms and improve social functioning. It might be noted that White et al. [50] found a reduction in negative symptoms in a feasibility trial of ACT for emotional dysfunction following psychosis. However, the very small sample size and apparent baseline differences makes it difficult to draw strong conclusions from this finding.Figure 1 summarises the expected pathways of action for ACT. Acceptance and defusion processes are expected to lead to reduced preoccupation, conviction, distress and disruption to life associated with hallucinations and/or delusions resulting in a general improvement in positive symptoms. Increased commitment to valued action is expected to lead to adaptive behavioral change and a general improvement in negative symptoms. The net outcome of these changes will be observed in improvements in overall mental state and social functioning.

Our hypotheses are therefore that, compared to patients receiving equivalent clinician time, patients who receive ACT will show improvement in (a) overall mental state (incorporating positive, negative and general symptoms); (b) preoccupation, conviction, distress and disruption to life associated with positive psychotic symptoms; (c) social functioning, and (d) adaptive behavior. Outcomes will also be examined in relation to (e) service utilisation. We hypothesise that all changes will be achieved by the conclusion of therapy and maintained at six-month follow-up. The study will also examine hypothesised mechanisms of action, namely that within the ACT group (f) improvements in positive symptoms will be predicted by reductions in experiential avoidance, (g) improvements in negative symptoms will be predicted by increased commitment to action and (h) if any paradoxical increase in the number of symptoms are reported, this will be mediated by recovery style. Finally, we considered that cognitive impairments commonly seen in schizophrenia [51-53] could interfere in understanding abstract metaphors and retaining information in the ACT condition, thereby moderating outcomes. The inclusion of several cognitive measures as covariates, including verbal memory, abstract reasoning and premorbid IQ, will allow us to examine the role of cognitive functioning on outcome.

## Methods/Design

### Trial design

The study is a prospective, single (rater)-blind, RCT using parallel group comparison design involving the intervention, ACT, and a comparison condition, Befriending. Both types of intervention are provided by all therapists involved in the trial. Participants continue their usual treatment during the course of their participation in the trial. Assessments take place prior to commencing therapy (baseline), at the completion of therapy (endpoint), and at 6 months follow-up. The study is being conducted in compliance with the Helsinki Declaration and has been approved by the following governing ethics committees: Alfred Hospital Ethics Committee (273/09); Austin Health Human Research Ethics Committee (H2009/03570); Eastern Health (E13/1011); La Trobe University Human Ethics Committee (07–160); Melbourne Health Mental Health Research and Ethics Committee (2007.39); Mercy Health & Aged Care Human Research Ethics Committee R08/34 W); Peninsula Health Human Research & Ethics Committee (HREC/11/PH/4); Southern Health (now Monash Health) Human Research Ethics Committee A (08084A); St. Vincent’s Hospital Human Research Ethics Committee (045/08); and The Melbourne Clinic Research Ethics Committee (185). Written informed consent is required from all participants.

### Sample

#### Selection criteria

Inclusion criteria include: (a) age between 18 and 65 years inclusive; (b) a current diagnosis of schizophrenia or schizoaffective disorder, according to the *Structured Clinical Interview for DSM-IV-TR Axis I Disorders*[54]; (c) current residual hallucinations or delusions which are associated with significant distress or disability (a score of 4 or greater on items P1 and/or P3 of the Positive and Negative Syndrome Scale (PANSS - [55]); (d) these symptoms have been present continuously over the past six months; and (e) have been on antipsychotic medication at doses within the therapeutic range over the past six months. Exclusion criteria are: (f) any neurological disorder that may affect cognitive function; (g) insufficient conversational English for meaningful participation; (h) having an IQ less than 70, as estimated by the Wechsler Test of Adult Reading (WRAT - [56]); (i) any change in antipsychotic medication within the previous eight weeks or planned at the time of intake; (j) currently receiving other formal psychological treatment.

#### Sample size

We determined that a sample of 53 participants per treatment arm would enable endpoint between group effects for overall mental state of *d =* 0.55 or greater to be detected with 80% power (α = 0.05). The effect size chosen is a little lower than the *d = 0.60* reported in the TORCH [38] and Gaudiano and Herbert [36] trials for overall mental state.

#### Recruitment and randomisation

Participants are recruited from public mental health services and private providers including non-government psychiatric disability rehabilitation services in metropolitan Melbourne, Australia. Potential participants are identified via clinician referral, supplemented by review of clinician case lists and advertising within services and print media to generate self-referrals. Participants who provide written informed consent undertake an interview to complete the baseline assessment measures prior to randomisation and confirm eligibility.

The randomisation materials and procedure was prepared at study inception by an independent statistician who subsequently had no further involvement in the project. Randomisation is stratified by site of recruitment (9 sites) and recovery style (integration or sealing over). Within each of the 18 factorial combinations of site of recruitment and recovery style, a random permuted blocks procedure was used to generate a list of As and Bs. The procedure was implemented in Microsoft Excel, and a high quality random number generator was used to choose each random sequence of blocks, without replacement within each sequence. For each of the 18 groups a numbered pile of sealed opaque envelopes was prepared, each envelope containing a slip of paper marked ‘A (ACT)’ or ‘B’ (Befriending), in accord with the generated sequence. The printing on the slips is very faint, and slips were folded so that no hint of the A or B can be seen through the envelope even when held up to light. Using the envelopes in numbered order, for the appropriate group, gives an allocation of subjects to treatment A or B in accordance with CONSORT guidelines. The random number seed, and the 18 printed sequences of As and Bs, are sealed in double envelopes, with again, no hint of the contents being visible through the envelopes. Assignment of participants to conditions using these materials is undertaken by a research assistant who works independently of staff involved in the recruitment, assessment and management of participants in the study. Group allocation is revealed to the trial therapists and by letter to the participant and their primary treating clinician.

### Treatments

#### ACT

Participants randomised to the ACT condition are offered eight 50-minute sessions of ACT, delivered at weekly to fortnightly intervals within a time frame of around three months. We considered that an eight-session intervention would provide a more comprehensive treatment for our chronically affected sample than that offered in the two trials with acute inpatients, but would nevertheless remain much briefer than trials of CBTp, which average 20 sessions in length [35], and our combined ACT-CBT TORCH protocol of 15 sessions [38]. ACT is conducted according to a local manual which is largely based on the first edition of the ACT manual [29] but with recommended adaptations for psychosis [34,57]. The 8 sessions comprise an initial assessment session followed by 7 intervention sessions. The initial session includes an orientation to therapy and an assessment of symptoms and problems related to psychosis. During this session, the therapist also notes the participant’s degree of struggle with symptoms and motivation for change as basic indicators of the most useful starting point for subsequent interventions. For example, creative hopelessness, the traditional beginning to ACT, is likely to be most suitable for clients who have struggled with their symptoms and are highly motivated to change. For clients who are less motivated or ambivalent about therapy it may be more useful to start with a focus on values and goals. While guidelines are provided, the therapist is expected to tailor the components according to the needs of the participant as identified in the assessment phase. The order in which these intervention components are delivered is also left to the therapist’s judgment. Participants are provided with folders for handouts and sessions are recorded onto CDs for home review.

#### Befriending

Participants randomised to the control condition are offered eight 50-minute sessions of the Befriending intervention [58], a fully manualised treatment previously used as a control condition in trials of psychological intervention in schizophrenia (e.g., [59]), including our TORCH trial [38]. Befriending involves engaging in conversation about everyday topics, whilst explicitly avoiding discussion of symptoms, problems or emotive issues. It has been shown to provide the same amount of therapist engagement and expectancy as CBT and to have similar drop-out rates [60].

In both conditions, therapy is provided by clinical psychologists with experience of psychological interventions in schizophrenia and with additional training in ACT and Befriending. They attend weekly peer supervision led by JF with SH providing advice and assistance in dealing with complex ACT-related issues through monthly supervision via videoconferencing. Medication, case management and other aspects of treatment continue to be managed by local services. At the end of treatment, a summary report is provided to the primary treating clinician.

### Measures

#### Outcome measures

##### Overall mental state

Overall impact upon mental state is measured by the Positive and Negative Syndrome Scale (PANSS - [55]), a widely-used, comprehensive interview-based measure of schizophrenia symptomatology which consists of seven items measuring positive symptoms, seven items measuring negative symptoms and 16 items measuring general symptoms. We will report overall mental state (as measured by the total score) along with subscale scores.

##### Positive symptoms

The Psychotic Symptom Rating Scales (PSYRATS - [41]) is a semi-structured interview designed to assess the severity of an individual’s auditory hallucinations and delusions across a range of physical and psychological dimensions. We use items from the PSYRATS to assess the severity of hallucinations and delusions according to the specific dimensions of interest to the study which include preoccupation, conviction, distress and disruption to life. In order to be able to control for the presence and frequency of symptoms, the frequency dimension of the PSYRATS auditory hallucinations subscale and the amount of preoccupation dimension of the PSYRATS delusions subscale is also be assessed. This scale has good psychometric properties and excellent inter-rater reliability across both scales is excellent, ranging from 0.788-1.00 for hallucinations and 0.884 – 1.00 for delusions [41].

Delusions are further assessed using the Peters Delusions Inventory (PDI - [43]). This self-report measure assesses delusional thinking more broadly and has been found to be a reliable and valid of delusional thinking in the general population and to discriminate between clinical and non-clinical samples on conviction, preoccupation and distress [43]. Participants are asked to indicate the presence or absence of 21 delusional beliefs (e.g., “Do you ever feel as if people are reading your mind?”) giving a total score of 0–21. Each delusional belief endorsed is then rated on dimensions of preoccupation, conviction and distress. The total number of items endorsed on the PDI will be used to assess for any paradoxical increases in symptom reporting.

##### Social functioning

Impact on overall social functioning is assessed with the Social Functioning Scale (SFS - [61]). This is a comprehensive interview-based assessment for individuals with schizophrenia that assesses seven areas of functioning essential to living in the community (social engagement, interpersonal behavior, prosocial activities, recreation, independence and employment). It has been shown to be valid, reliable, and sensitive to change [61]. This is completed on the basis of interview with the participant and discussion with their case manager or alternative informant. The Sheehan Disability Scale (SDS - [62]) is a 3-item self-report instrument measuring impairment and functional disability due to psychiatric symptoms across key domains of work/education, social life and family life/home responsibilities. Responses are measured on a 10-point partially anchored visual analogue scale from 0 (not at all) to 10 (extremely) and assessed over the last month.

##### Adaptive behavior

Direct impact on behavior is assessed using a time budget measure for schizophrenia [63] which calculates the level of adaptive activity undertaken by the participant over a representative week on the basis of a detailed interview. Level of adaptive activity is rated from 0: nothing – lying thinking sleeping sitting etc. to 4: time period filled with a variety of demanding independent activities requiring significant motivation and planning and with some variation in tasks, e.g., work. Four time blocks per day are rated over 7 days with the total weekly score ranging from 0 – 112. It has been used successfully with schizophrenia, with good reliability and validity [63]. The specific hypothesis that treatment will moderate the impact of negative symptoms will be assessed using the PANSS Negative Syndrome subscale.

##### Service utilisation

Service utilisation during the follow-up period will be compared with baseline rates of service utilisation using a subset of self-report questions extracted from instrumentation as used in the previous (2007) Australian National Survey of Mental Health and Wellbeing. Forms of service use include both psychiatric hospital admissions (number and days) and consultations with mental health service providers (number and length). This broader measure of service use is used in preference to rehospitalisation rates alone, due to the relatively low baseline rate of readmission in a chronic sample. Indeed, Gumley et al. [64] estimate a sample size of over 400 participants would be required to detect changes in readmission rates, which was not feasible in this study.

#### Process measures

##### Acceptance and commitment

The 16-item Acceptance and Action Questionnaire (AAQ - [65]) is used in order to measure the hypothesised therapy mechanisms of (a) increasing the degree of acceptance of psychotic experiences as opposed to attempted suppression and experiential avoidance, and (b) increasing commitment to valued action. Like the more commonly used 9-item AAQ, this version has single factor structure. It was chosen on the basis of the authors’ view ([58], p. 563) that in a research context the additional items were likely to confer greater sensitivity to change. (The present study was devised prior to publication of the AAQ II [66]). Internal consistency of the 16-item version was not reported however it correlates .89 with the 9-item version which has a Cronbach alpha of .70. Extensive convergent and divergent validity has been reported with versions of the measure widely used in research on the mechanisms of ACT [32,65]. As this measure has not been previously applied to psychosis, our group has previously developed a measure for auditory and command hallucinations that directly taps each construct (the Voices Acceptance and Action Scale – VAAS), with promising initial results and acceptable psychometric properties [67]. The VAAS is used in addition to the AAQ for participants experiencing auditory hallucinations.

##### Recovery style

The Recovery Style Questionnaire (RSQ - [68]) is used to assess the degree to which participants ‘integrate’ their illness, acknowledging their illness experiences with interest and curiosity (e.g., “I can see positive aspects to my illness”; “My illness is part of me”), as opposed to ‘seal over’ whereby participants seek to encapsulate their psychosis as separate from themselves and express disinclination for any exploration of their illness experiences (e.g., “My illness has had little effect on my life”; “I’m not really interested in my illness”). This self-report scale has 39 items; higher scores indicate greater sealing over. The RSQ has been evaluated as a valid measure of recovery style according to the original concept [49,69]. It has been found to have high test-retest reliability (Spearman 4 = 0.81) and an acceptable Cronbach’s alpha of 0.73 [68].

#### Covariates

##### Premorbid IQ

The WTAR is administered as a test of premorbid IQ. Participants are asked to read a list of 50 words that have atypical grapheme to phoneme translations (e.g., “liaison”, “ paradigm”) and are scored according to accuracy of pronunciation (raw score range 0–50). WTAR raw scores are standardised based on age then converted to the predicted WAIS-III IQ. While predicted IQ scores will be reported descriptively, these are associated with very large confidence intervals; standardised scores will therefore be used for analyses due to their greater precision.

##### Abstract verbal reasoning

The Similarities subscale of the Wechsler Adult Intelligence Scale III (WAIS-III) [70] is administered to assess abstract verbal reasoning in relation to concept formation. The subscale consists of 19 items: participants are asked to describe how two given things are alike (e.g., “table and chair”, “poem and statue”). Raw scores are converted to age-adjusted scaled scores using the normative data provided in the manual.

##### Verbal memory

The Story Memory and Story Recall subtests of the Repeatable Battery for the Assessment of Neuropsychological Status (RBANS) [71] are used to assess immediate and delayed memory. Story Memory involves reading a brief story and asking the participant to repeat it back immediately. It is scored over two trials (total score 0–24). Story Recall involves free recall of the story after a delay of 15–20 minutes (total score 0–12). Raw scores will be converted to z scores for analyses using normative data for each participant’s age group [72].

##### Antipsychotic medication

Antipsychotic medication changes and dosages are tracked at each assessment point and will be converted to chlorpromazine-equivalent dosages [73]. Chlorpromazine-equivalent dosages will be compared between groups in order to verify whether groups are equivalent, and correlations will be examined between dosage changes during the study period and clinical outcomes in order to exclude the possibility that medication changes are responsible for outcome.

#### Therapy evaluation

At the end of their post-therapy assessment, participants are asked to complete the Client Satisfaction Questionnaire-8 (CSQ-8 - [74]) to assess the acceptability and experience of therapy. As well, four additional questions give participants an opportunity to provide more specific feedback about their experience of therapy. These include ratings of emotional response and problem improvement with respect to problems related to psychosis and qualitative feedback related to therapy gains and disappointments. We expect that therapy will be rated positively by a majority of patients.

#### Treatment fidelity

Treatment sessions are audio recorded and a stratified random sample of audio files will be rated by an independent assessor for compliance with therapy condition. The assessor, blinded to treatment condition, will be required to assign each audio file to either ACT or Befriending. Because no validated scale currently exists to measure therapist adherence to ACT guidelines more specifically, an ACT fidelity scale is developed in the initial phase of the trial. The Befriending Treatment Integrity Measure [58] is used to assess the quality of Befriending sessions and to ensure that ACT sessions do not include Befriending techniques.

### Primary outcome

The primary outcome is overall mental state as measured using the PANSS total score. PANSS subscale scores for positive, negative and general scales will also be reported.

### Secondary outcomes

Secondary outcomes are: the PSYRATS subscales (preoccupation, conviction, distress, and disruption to life) adjusting for the presence and frequency of symptoms; the SFS; and service utilisation.

### Procedure

Assessments are conducted by research assistants trained in administration of the measures at baseline (prior to randomisation), at the end of therapy approximately three months following baseline (endpoint), and at six months following the end of therapy (follow-up). Outcome measures, process measures and medication are assessed at all three time points except for service utilisation which is administered only at baseline and follow-up. Cognitive measures are done where possible at Time 1 or, if necessary, at a later time point.

### Blindness

Considerable efforts are made to ensure that the blindness of raters is maintained. Offices, data storage and travel logs of raters and therapists are kept separate. Participants and clinical staff are regularly reminded not to divulge details of their therapy to the raters and a reminder sign placed prominently in front of the participant during assessments. Blindness is assessed after endpoint and follow-up assessments by asking raters classify participants into treatment condition and to indicate their level of confidence. Breaches in blindness are recorded and addressed by changing the rater wherever possible.

### Statistical analysis

The primary focus of the analysis is differential changes in the ACT group versus Befriending from baseline to endpoint, which will be analysed within the framework of Treatment Group (ACT vs. Befriending) by Measurement Occasion (baseline, endpoint, follow-up) design. Analyses will be undertaken using mixed-model repeated measures (MMRM) which is the recommended method for examining clinical trial data [75]. Primary evidence of the efficacy of ACT will be a significant two-way interaction demonstrating greater change in outcome measures in the ACT group from pre- to post-therapy. Baseline to follow-up interactions with treatment group will also be examined. Where there are significant group x time interactions, planned contrasts will compare changes from baseline under each intervention at endpoint and follow-up.

Intention-to-treat (ITT) analysis [76] will be used. MMRM is well-suited to ITT analyses because this approach uses all available information from subjects as randomised to produce ITT estimates of treatment effects under only mild assumptions concerning the nature of withdrawal [75]. Most importantly, subjects with incomplete data are not discarded and missing data are not replaced with unprincipled estimated values or observations carried forward [77]. This approach is regarded as the most appropriate method of analysing RCT data by the highest quality journals, which have adopted the CONSORT standard (see, e.g., [78]). However, if data are grossly non-normal and cannot be successfully transformed, non-parametric methods will be used.

The mediational impact of pre to post AAQ, RSQ and medication dose change will be assessed using the Sobel test [79], arguably the most powerful current method of detecting indirect effects [80]. This test assesses the statistical significance of the product of the coefficients for treatment-mediator and mediator-outcome effects. A bootstrapped multivariate extension of the Sobel test described by Preacher and Hayes [81,82] will be used, which is suitable for non-parametric data, and allows us to examine both the total indirect effect and the individual effect of each specified mediator, controlling for the other.

## Discussion

This protocol describes the first RCT of ACT in chronic medication-resistant psychosis. It is also the first RCT of ACT in psychosis to use an active comparison group, and be designed to fully meet CONSORT criteria. It thus rises to the challenge of critics both of ACT trials, such as Öst [83], who point out the scarcity of well-controlled trials of the therapy across many client groups, and critics of CBTp trials who note variable trial quality [13,15] and the negative correlation between trial quality and strength of outcome [13,84]. If successful, it will be the most definitive trial to date of ACT for people living with psychosis.

ACT for psychosis holds promise as a therapy that may compare favourably with CBTp in outcomes, uptake by patients, treatment duration and ease and costs of training. By adopting an alternative therapeutic method, ACT may show greater applicability to psychosis than CBT, or may prove to be more suitable for some patients who currently fail to respond to CBT. Additionally, from its explicit emphasis on reducing behavior in response to symptoms and promotion of behavior in line with values, ACT may be more successful than CBT in reducing negative symptoms and symptom-related disability [50]. In addition, its grounding of treatment in the individual’s values is likely to provide an approach which is highly acceptable to patients and consistent with the recovery framework promoted by consumers and adopted by mental health services in many countries. Furthermore, by following a set manualised structure, rather than relying upon the complex and lengthy process of belief modification, therapy can be much briefer (8 sessions ACT *vs*. an average of 20 sessions CBTp) and potentially practiced by a wider range of clinicians. ACT provides an approach which is more cost-effective and more readily manualised than existing psychological treatments: this may lead to it being more widely disseminated into routine practice, and ultimately lead to greater availability of effective treatment to consumers.

## Abbreviations

AAQ: Acceptance and action questionnaire; ACT: Acceptance and commitment therapy; BPRS: Brief psychiatric rating scales; CBT: Cognitive behavior therapy; CBTp: Cognitive behavior therapy for psychosis; CSQ-8: Client satisfaction questionnaire-8; DSM-IV_TR: Diagnostic and statistical manual of mental disorders 4^th^ edition, text revision; IQ: Intelligence quotient; ITT: Intention to treat; MMRM: Mixed-model repeated measures; PANSS: Positive and negative syndrome scale; PDI: Peter’s delusions inventory; PSYRATS: Psychotic symptom rating scales; RBANS: Repeatable battery for the assessment of neurological status; RCT: Randomised controlled trial; RSQ: Recovery style questionnaire; SDS: Sheehan disability scale; SFS: Social functioning scale; TORCH: Treatment of resistant command hallucinations; VAAS: Voices acceptance and action scale; WAIS: Wechsler adult intelligence scale; WTAR: Wechsler test of adult reading.

## Competing interests

The authors declare that they have no competing interests.

## Authors’ contributions

All authors participated in the design of the trial. All authors read and approved the final manuscript.

## Pre-publication history

The pre-publication history for this paper can be accessed here:

http://www.biomedcentral.com/1471-244X/14/198/prepub
